# Effect of novel anti-tumor and anti-angiogenesis drug taurolactone on angiogenic factor AGGF1 and angiogenesis mimicry in patients with hepatocellular carcinoma

**DOI:** 10.1186/s12885-024-12356-w

**Published:** 2024-05-21

**Authors:** Shaoping Liu, Yinzhi Wei, Lei Nie, Ze Tang, Qi Lu, Qun Liang

**Affiliations:** 1 Department of General Practice, Huangshi Central Hospital, Affiliated Hospital of Hubei Polytechnic University, No.43 Wuhan Road, Huangshigang District, Huangshi, 435000 Hubei China; 2 Department of Abdominal Tumor Surgery, Hubei Province Cancer Hospital, Wuhan, China; 3 Department of Abdominal Oncology, Huangshi Central Hospital, Affiliated Hospital of Hubei Polytechnic University, Huangshi, China; 4 Department of HepatobiliarySurgery, Huangshi Central Hospital, Affiliated Hospital of Hubei Polytechnic University, Huangshi, China

**Keywords:** Taurolactone, Hepatocellular carcinoma, Angiogenic factor AGGF1, Angiogenesis mimicry

## Abstract

**Objective:**

Our study was to investigate the impact of taurolactone, a novel anti-tumor and anti-angiogenic drug, on AGGF1, an angiogenic factor, and angiogenesis mimicry in patients diagnosed with hepatocellular carcinoma (HCC).

**Methods:**

A total of 120 HCC patients were enrolled from the Department of Oncology and Hepatobiliary Surgery at our hospital between May 2021 and December 2022. HCC diagnoses were confirmed through imaging or tissue biopsy for all patients. The age of patients ranged from 37 to 72 years, with an average age of 64.29 ± 4.58 years. These participants were divided equally into two groups: the control group and the observation group, each consisting of 60 individuals. While the control group received standard drug treatment, the observation group was administered taurolactone treatment. Before being included in the study, all participants or their legal representatives provided signed informed consent. Patient demographic information was collected through a questionnaire survey. ELISA was used to measure the levels of VEGF and AGGF1 in patients following treatment. Western blot was applied to assess the protein expression of PDGF, Angiopoietin, and AGGF1. MRI imaging technology was utilized to assess the perfusion characteristics of tumor blood vessels in patients. Tumor vessel density was compared between patients using ultrasonography. We also conducted a comparison between the two groups in terms of progression-free survival and overall survival.

**Results:**

General patient information between the two groups showed no significant differences (*P* > 0.05). Of note, the observation group exhibited greatly lower levels of VEGF and AGGF1 compared to the control group (*P* < 0.05). Moreover, the levels of PDGF, Angiopoietin, and AGGF1 protein expression were significantly reduced in the observation group compared to the control group (*P* < 0.05). In terms of tumor perfusion, the observation group displayed lower average and maximum perfusion volumes in tumor blood vessels compared to the control group (*P* < 0.05). Additionally, the observation group demonstrated delayed peak times and arrival times of tumor blood vessels in comparison to the control group (*P* < 0.05). Furthermore, the density of tumor blood vessels was notably lower in the observation group compared to the control group (*P* < 0.05). Patients in the observation group had longer progression-free survival and overall survival than the control group (*P* < 0.05).

**Conclusion:**

In HCC patients, our study highlighted the potential efficacy of taurolactone treatment as it effectively inhibited angiogenic factors and angiogenesis mimicry, ultimately leading to an improved prognosis for these patients.

**Supplementary Information:**

The online version contains supplementary material available at 10.1186/s12885-024-12356-w.

## Introduction

Hepatocellular carcinoma (HCC) is a highly aggressive and challenging cancer with a global impact [1]. One of its defining characteristics is its invasive nature, making it notoriously difficult to treat, often accompanied by complex angiogenesis processes. Angiogenesis, both in its natural and abnormal forms, involves the formation and progression of new blood vessels, which are crucial for supplying tumors with vital oxygen and nutrients. Consequently, angiogenesis significantly influences the proliferation and metastasis of tumors [2–4]. Addressing the clinical challenges posed by HCC, particularly in its advanced stages, has been a longstanding concern. In recent years, anti-angiogenesis therapy has emerged as a promising approach to cancer treatment, drawing significant attention. The fundamental principle behind this strategy is to limit the nutrient supply of tumor by disrupting its angiogenesis processes, thus inhibiting its growth and spread [5, 6]. Research has found that taurine can kill tumor cells by activating macrophages, inhibiting tumor cell proliferation, inhibiting tumor angiogenesis, and directly inhibiting tumor growth [5, 6].The research and application of antiangiogenic drugs have achieved remarkable clinical results in various cancer types. Taurolactone, a novel and promising drug with potential anti-cancer and anti-angiogenic properties, has garnered significant attention. Taurine, an organic compound containing sulfur, has demonstrated anti-angiogenic and anti-tumor properties in various disease models [7]. Nevertheless, additional research and elucidation are required to fully understand the potential impact and underlying mechanisms of this treatment approach for HCC. This study aimed to investigate the therapeutic potential of taurolactone in individuals harboring HCC, with a specific focus on its effects on the angiogenic factor AGGF1 and angiogenesis mimicry. AGGF1’s role and regulation mechanism in HCC, as an important regulator of angiogenesis, have garnered significant interest but remain incompletely understood. Through a systematic experimental design and rigorous data analysis, our goal was to investigate the taurolactone’s capacity to disrupt AGGF1 and angiogenesis mimicry. Additionally, we sought to assess the therapeutic advantages it may offer to individuals diagnosed with HCC. Our research may contribute to a more holistic comprehension of the potential mechanisms underlying anti-angiogenesis treatment for HCC, establishing a strong scientific basis for future clinical applications.

## Materials and methods

### General information

Between May 2021 and December 2022, a total of 120 individuals diagnosed with HCC were recruited as participants at our hospital. Among them, 85 cases were identified as primary liver cancer, 17 cases as cholangiocarcinoma, and 18 cases as hepatocellular steatosis carcinoma. The diagnosis of HCC was confirmed through imaging or tissue biopsy. Patients’ ages ranged from 37 to 72 years, with an average age of 64.29 ± 4.58 years. The study included 72 male patients and 48 female patients. These patients were randomly allocated into two groups: the control group and the observation group, each consisting of 60 cases. Informed consent was obtained from either the patients themselves or their legal guardians prior to their participation in the study.

1. The control group was given standard drug treatment: magnesium isoglycyrrhizinate injection [ZhengdaTianqing Pharmaceutical Group Co., LTD., Sinopath approval number H20051942, specification 10mL: 50 mg (based on magnesium isoglycyrrhizinate)], 150 mg each time, dissolved in 0.9% sodium chloride injection 250mL, intravenous infusion, once a day; Reduced glutathione for injection (Shanghai FudanFuhua Pharmaceutical Co., LTD., National Drug Approval number H20060450, specification: 0.9 g), 1.8 g each time, add 0.9% sodium chloride injection 100mL, intravenous infusion, once a day. Chemotherapy was administered every 2 weeks for 8 weeks.
2. The observation group was given taurine lactone treatment: on the basis of the treatment of the control group, taurine (Shanghai ShangyaoXinyi Pharmaceutical Co., LTD., Sinopma approval number H1999942) was added for treatment, 400 mg/ time, once/day. Continuous treatment for 6 weeks.

### Inclusion criteria

Patients were considered eligible for inclusion in the study if they met the following criteria: Age of at least 18 years, regardless of gender; Diagnosis of HCC based on clinical assessment and imaging examinations such as CT or MRI; General health suitable for treatment and laboratory examinations; Liver function tests, such as liver function enzyme levels, bilirubin, and albumin levels, within the acceptable range; Capacity to comprehend the study and willingness to sign the informed consent form for research participation.

### Exclusion criteria

Patients meeting the following criteria were excluded: Presence of other malignant tumors or severe medical conditions that could potentially interfere with the interpretation of research results. Severe cardiovascular conditions such as unstable chest pain and cardiac insufficiency; Severe central nervous system disorders that could affect cooperation during treatment or the feasibility of evaluation; Known severe allergies or negative responses to taurolactone or similar medications.Concurrent participation in other experimental drug treatments or interventional clinical trials; Refusal to provide informed consent for participation in the study.

### Ethical information

Prior to the study, the relevant Ethics Review Committee at our hospital approved the research protocol. Moreover, all research participants provided informed consent upon their enrollment. All procedures and activities undertaken throughout this research complied with ethical norms and regulations.

### ELISA analysis

Blood samples were collected from participants using serum separation tubes. These samples were allowed to coagulate for 30 min and subsequently subjected to centrifugation. The resulting serum samples were promptly stored at a temperature of -80 °C. For the quantification of VEGF and AGGF1, a commercially available human quantitative enzyme-linked immunosorbent assay (ELISA) kit (R&D Systems, Minneapolis, Minnesota, USA) was employed. The assays were conducted in duplicate, in accordance with manufacturer’s guidelines.

### Western blot analysis

Following the preparation of plasma lysates, the samples were separated using SDS-PAGE and then transferred onto a PVDF membrane. The membrane was subsequently blocked with 5% skimmed milk at room temperature for 60 min. After washing the membrane, the primary antibody was incubated at 37℃ for 2 h, followed by the secondary antibody, which was also incubated at the same temperature for 1 h. The blots were cut prior to hybridization with antibodies during blotting. Primary antibodies used included PDGF, Angiopoietin, and AGGF1 rabbit antibodies, along with radish peroxidase coupled anti-GAPDH (Kangchen Biotechnology). Horseradish peroxidase-conjugated secondary antibodies, specifically goat anti-rabbit and anti-mouse PDGF, Angiopoietin, and AGGF1 were employed. Protein visualization was achieved using the SuperSignal West Pico Chemiluminescence Substrate Kit (Pierce Biotechnology), following the manufacturer’s guidelines after the washing process. Density analysis was conducted using AlphaImager 2200 analysis software.

### MRI imaging technology for perfusion parameter evaluation of tumor blood vessels in patients

In patients with liver cancer, the perfusion parameters of tumor blood vessels were assessed using dynamic contrast-enhanced magnetic resonance imaging (DCE-MRI). Patient were positioned on the MRI table and to ensure minimal movement during the examination. MRI scans were conducted to obtain images. The tumor’s location and morphology were determined through magnetic resonance imaging. After capturing the initial image, patients received an intravenous injection of a contrast agent. Subsequently, a sequence of dynamic images was captured and analyzed to compute diverse perfusion parameters. These included average perfusion, maximum perfusion, peak time, and time of arrival. Average perfusion denotes the mean blood circulation within the tissue during a specific time frame. Maximum perfusion refers to the highest volume of blood flow occurring within a specific time frame in the tissue. Peak time refers to the duration between the administration of the contrast agent and the point of maximum contrast agent perfusion. It reflects the speed at which the contrast agent reaches the tumor, providing insights into its angiogenesis and hemodynamic characteristics. Time of arrival indicates when the contrast agent enters the tissue or tumor, aiding in comprehending the rate at which the contrast agent reaches distinct tissue regions, which is valuable for assessing the tumor’s blood flow.

### Detection of vascular density

To initiate the fundamental ultrasound examination and acquire standard ultrasound image of the tumor’s location and morphology, patients received an intravenous injection of an ultrasound contrast agent. Following the administration of the ultrasound contrast agent, the process of obtaining contrast ultrasound images commenced. Upon entering the tumor’s vascular system, it generated a vivid signal, enabling doctors to visually examine the blood vessel arrangement within the tumor. Doctors analyzed the contrast-enhanced ultrasound images to assess the density and arrangement of blood vessels within the tumor. Quantitative analysis of the regions displaying intense signals in the images allowed for an estimation of the tumors’ vascular density.

### Survival analysis

We conducted survival analysis to assess the overall survival and progression-free survival rates of liver cancer patients in both groups. Overall survival is defined as the duration from the initial diagnosis or treatment to the patient’s demise or the most recent follow-up. Progression-free survival is defined as the duration between the initiation of treatment and disease progression or patient mortality. The treatment initiation time and the time of disease progression or mortality were documented for each patient.Complete response (CR): All target lesions disappear, no new lesions appear, and tumor markers remain normal for at least 4 weeks. Partial response: The maximum sum of diameters of the target lesion is reduced by ≥ 30% and maintained for at least 4 weeks. Stable disease (SD): The sum of the maximum diameters of the target lesion decreases below PR or increases below PD. The sum of maximum diameters of target lesions in disease progression (PD) increases by at least 20%, or new lesions appear.CR + PR = OR - Objective relief. Reduction does not reach PR (baseline lesion total length and diameter reduction ≥ 30%) or increase does not reach PD (baseline lesion total length and diameter increase ≥ 20% or new lesions appear, or/and non target lesion progression), one or more non target lesions and/or biomarkers are abnormal.

### Statistical analysis

This study used SPSS20.0 statistical software; The measurement data is represented by “mean ± standard deviation” ($\overline x$± s), inter group comparisons are performed using one-way ANOVA, and inter group comparisons are performed using t-test. The counting data is expressed as a percentage (%), and inter group comparisons are made using χ^2^ analysis. *P* < 0.05 represents a statistically significant difference.

## Results

### Comparison of patient demographics

In the analysis of patient demographics, it was observed that the control group exhibited a male-to-female ratio of 39:21. The average age of the participants in this group was 63.37 ± 5.22 years, with an average BMI of 23.51 ± 1.88 kg/m^2^. Among the patients, there were 8 cases classified as TNM stage I, 34 cases as TNM stage II, and 18 cases as TNM stage III, with 17 cases exhibiting lymph node metastasis, 19 cases having a history of smoking, 11 cases following regular smoking patterns, and 32 cases with a history of alcohol consumption. In contrast, the observation group consisted of 34 males and 26 females, with an average age of 65.19 ± 4.78 years and an average BMI of 22.65 ± 2.01 kg/m^2^. In this group, there were 10 cases classified as TNM stage I, TNM 31 cases as stage II, 19 cases as TNM stage III, with 21 cases having a history of smoking and 29 cases with a history of alcohol consumption. Importantly, there were no discernible distinctions in the overall demographic data between the two groups (*P* > 0.05). (Table 1)

**Table 1:** Statistics of patient demographics

Index	Control group (*n* = 60)	Observation group (*n* = 60)	T value /χ^2^value	*P* value
Gender (male: female)	39: 21	34: 26	3.014	0.318
Age (years)	63.37 ± 5.22	65.19 ± 4.78	5.449	0.226
BMI(kg/m^2^)	23.51 ± 1.88	22.65 ± 2.01	6.218	0.503
TNM staging				
I(%)	8(13.33%)	10(16.67%)	2.664	0.667
II(%)	34(56.67%)	31(51.67%)		
III(%)	18(30.00%)	19(31.67%)		
Lymph node metastasis				
Yes (%)	17(28.33%)	20(33.33%)	5.211	0.109
No (%)	43(71.67%)	40(66.67%)		
Smoking history (%)	19(31.67%)	21(35.00%)	4.216	0.527
Drinking history (%)	32(53.33%)	29(48.33%)	2.007	0.664

### ELISA detection of VEGF and AGGF1

Following treatment, ELISA was utilized to measure the concentrations of VEGF and AGGF1 in the patients. The observation group exhibited significantly lower levels of both VEGF and AGGF1 than the control group (*P* < 0.05). (Table 2)

**Table 2:** ELISA analysis

Groups	VEGF(pg/ml)	AGGF1(pg/ml)
Control group (*n* = 50)	215.38 ± 22.18	144.26 ± 17.55
Observation group (*n* = 50)	179.33 ± 15.25	95.18 ± 10.22
*T value*	10.583	12.669
*P value*	0.001	0.001

### Western blot analysis of PDGF, Angiopoietin, and AGGF1 protein expression

**Fig. 1:**
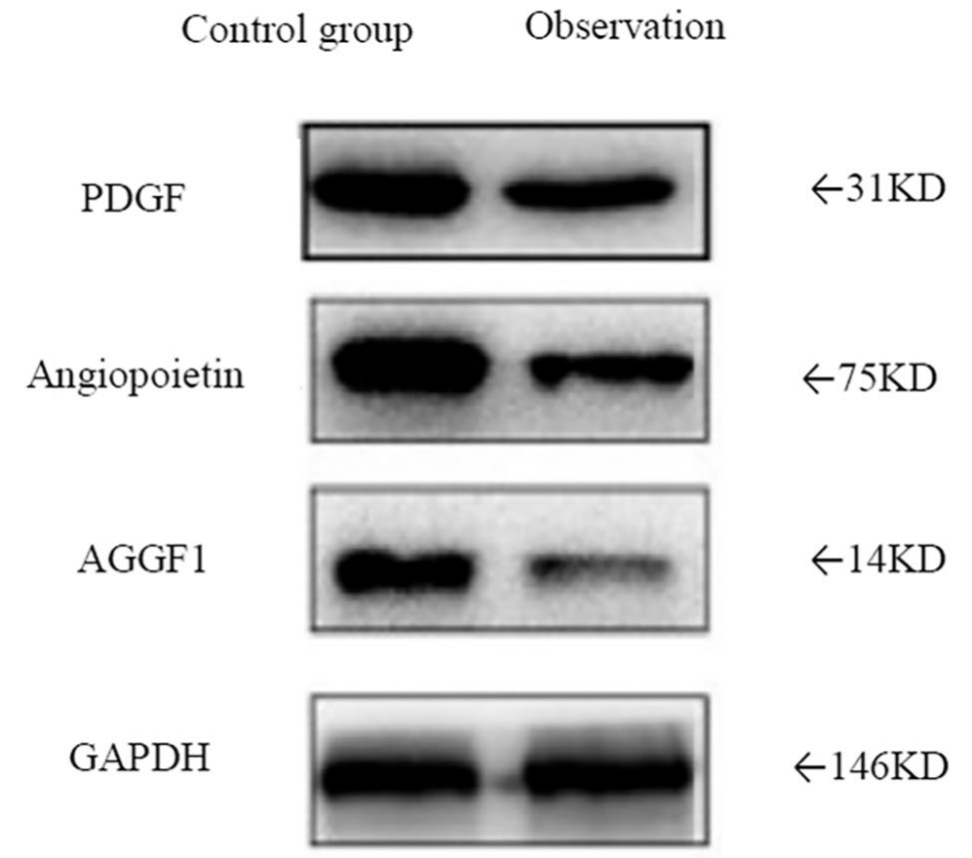
Western blot analysis of PDGF, Angiopoietin, and AGGF1 protein expression. Note: The levels of PDGF, Angiopoietin, and AGGF1 protein expression were notably reduced in the observation group compared to the control group

The levels of PDGF, Angiopoietin, and AGGF1 protein expression were notably reduced in the observation group compared to the control group (*P* < 0.05). (Fig. 1; Table 3)

**Table 3:** Protein expression of PDGF, Angiopoietin and AGGF1

Group	PDGF	Angiopoietin	AGGF1
Control group (*n* = 50)	1.95 ± 0.13	2.01 ± 0.18	1.98 ± 0.18
Observation group (*n* = 50)	1.13 ± 0.04	1.06 ± 0.02	1.08 ± 0.03
*T value*	15.226	9.304	12.119
*P value*	0.001	0.001	0.001

### Analysis of tumor vascular perfusion parameters

**Fig. 2:**
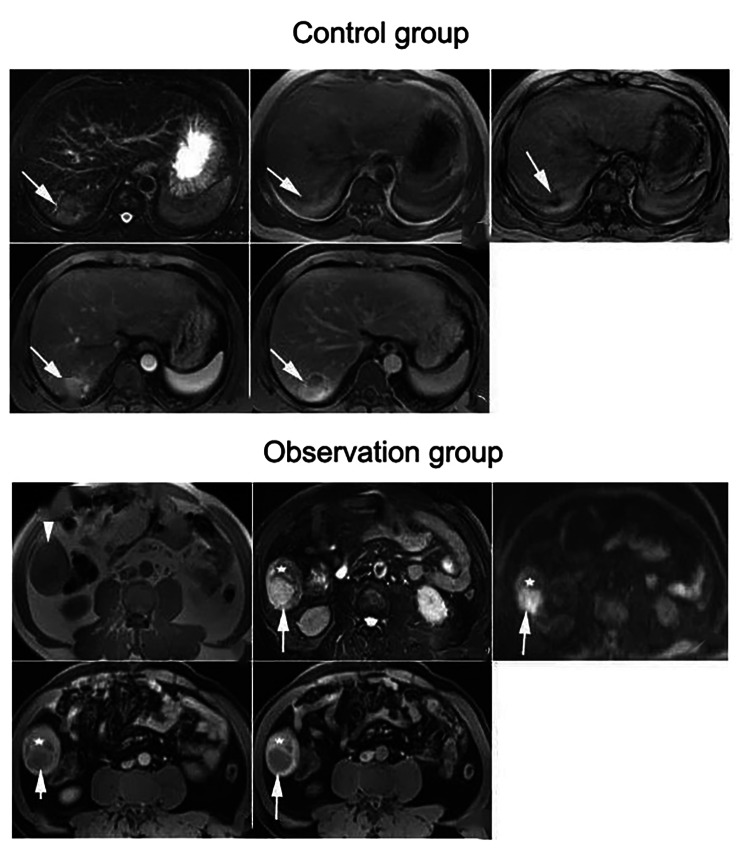
The perfusion characteristics of tumor blood vessels were assessed by MRI imaging technology. Note: Following treatment, compared to the control group, the observation group exhibited a decrease in both the average and maximum perfusion volume of tumor blood vessels, and showed prolonged peak time and duration for tumor blood vessels

MRI imaging technology was applied to assess the perfusion characteristics of tumor blood vessels. Compared to the control group, the observation group exhibited a decrease in both the average and maximum perfusion volume of tumor blood vessels (*P* < 0.05). Additionally, the observation group showed prolonged peak time and duration for tumor blood vessels when compared to the control group (*P* < 0.05). (Fig. 2; Table 4)

**Table 4:** Analysis of tumor vascular perfusion parameters

Groups	Average perfusion (mL/min/100 g)	Maximum perfusion (mL/min/100 g)	Peak time (s)	Time arrival (s)
Control group (*n* = 50)	128.65 ± 15.49	185.37 ± 17.15	15.38 ± 2.75	32.53 ± 3.69
Observation group (*n* = 50)	76.34 ± 10.22	144.29 ± 13.45	21.52 ± 3.25	43.47 ± 6.44
*T value*	11.447	10.315	13.688	9.143
*P value*	0.001	0.001	0.001	0.001

Captions on figures;

Control group: Axial MRI images of HCC patients in the left lobe of the liver. (a) T1 low signal was observed in the left lobe of the liver; (b) T2 high signal mass shadow, with multiple high signal (arrow) on T1WI and slightly high center and low signal ring (arrow) around it on T2WI, indicating intranodular hemorrhage; (c) Enhanced arterial phase mass; (d) Clarify the delay period.

Observation group: Axial MRI images of left liver HHCC patients. (a) T2 mixed with slightly high signal mass in the left lobe of the liver: (b) significantly uneven arterial phase with enhancement; (c) Clear portal vein phase with septa (arrows) visible.

### Tumor vascular density analysis

By means of ultrasound examination, the tumor vascular density in the observation group was evidently lower compared to the control group (*P* < 0.05). (Table 5)

**Table 5:** Analysis of tumor vascular density

Groups	Vascular density (individual / cm^2^)
Control group (*n* = 50)	54.18 ± 8.29
Observation group (*n* = 50)	38.23 ± 6.44
*T value*	9.113
*P value*	0.001

### Survival comparison

When comparing the progression-free survival time and total survival time between the two groups, it was found that patients in the observation group experienced longer progression-free survival time and overall survival times compared to those in the control group (*P* < 0.05). (Table 6)

**Table 6:** Comparison of overall survival and progression-free survival of patients

Groups	Progression-free survival (months)	Total survival (years)
Control group (*n* = 50)	5.43 ± 1.06	1.35 ± 0.16
Observation group (*n* = 50)	7.26 ± 1.38	1.87 ± 0.22
*T value*	12.377	14.156
*P value*	0.001	0.001

## Discussion

HCC is a malignancy characterized by its ability to stimulate angiogenesis, facilitating the acquisition of an adequate blood flow through the formation of new blood vessels [8, 9]. This process is commonly referred to as tumor angiogenesis and typically involves two primary mechanisms: angiogenesis, the generation of new blood vessels, and vasculogenesis, the enlargement of existing blood vessels. Cancerous hepatocytes often release specific growth factors that can induce the development of new blood vessels, thereby promoting tumor growth and metastasis [10, 11]. Medications designed to combat tumors, particularly those targeting HCC, hinder the growth of tumors by impeding the process of angiogenesis [12]. The outcome for individuals with HCC typically depends on the extent of blood vessel formation within the tumor. Tumors that are highly vascularized tend to be more aggressive and difficult to treat [13]. Hence, ongoing research efforts aim to identify novel therapeutic approaches capable of disrupting the angiogenic processes associated with HCC, ultimately improving patients outcomes. Taurine, a novel medication with anti-tumor and anti-angiogenesis properties, holds significant promise in suppressing tumor growth and metastasis [14, 15]. Taurine functions as a multi-target medication against tumors, with a primary focus on impeding tumor growth through the inhibition of tumor angiogenesis. It achieves this by targeting specific signaling pathways linked to various growth factors. Taurolactone, primarily disrupts the capacity of cancer cells to stimulate the formation of new blood vessels, thereby diminishing the blood supply to tumors. The inhibition of tumor angiogenesis can result in tumor ischemia, ultimately causing tumor cell death or growth arrest.

This study examined the potential clinical significance of taurolactone, a novel drug renowned for its anti-tumor and anti-angiogenesis properties, particularly concerning angiogenic factor AGGF1 and angiogenesis mimicry in individuals diagnosed with HCC. In patients with HCC, our findings indicated that taurolactone effectively decreased the levels of VEGF and AGGF1, prominent angiogenesis factors. This discovery is of great significance, shedding light on the therapeutic mechanism of taurolactone in HCC. VEGF and AGGF1 serve as key regulators of tumor angiogenesis, playing a critical role in tumor growth [16, 17]. VEGF facilitates the proliferation and formation of new blood vessels, thereby ensuring the supply of oxygen and nutrients to tumors. Concurrently, AGGF1 also contributes significantly to the intricate process of angiogenesis. Hence, diminishing the presence of these elements can impede the formation of blood vessels within tumors, restricting their expansion and dissemination. By suppressing the expression or secretion of VEGF and AGGF1, taurolactone has the potential to enhance the therapeutic outcomes for individuals suffering from HCC. Reduced levels of VEGF and AGGF1 may result in a reduction in the number of blood vessels within tumors, consequently limiting the delivery of oxygen and nutrients to tumor cells.

The levels of PDGF (platelet-derived growth factor), Angiopoietin, and AGGF1 (hepatocyte growth factor) in protein expression showed a significant decrease in the observation group. This decrease offered valuable insights into the mechanistic action of taurolactone in HCC treatment. PDGF, a critical growth factor, plays a pivotal role in the proliferation and differentiation of smooth muscle cells and fibroblasts during angiogenesis, particularly in the generation of new blood vessels [18]. The observed decline in PDGF concentration in the observation group suggested that taurolactone could disrupt the angiogenesis of HCC by suppressing the expression or secretion of PDGF. This inhibition may hold potential benefits for reducing angiogenesis and limiting tumor growth. Angiopoietin, a cluster of proteins, has a crucial function in both angiogenesis and the preservation of vascular stability [19]. It contributes to the regulation of blood vessel stability through interaction between vascular endothelial cells and surrounding tissues. The decline in Angiopoietin levels in the observation group may raise the possibility of vascular instability, which could disrupt the angiogenesis process in HCC and restrict tumor growth. AGGF1, recognized for its involvement in cellular growth and angiogenesis [20], functions as a growth factor. The decline in AGGF1 concentration among patients in the observation group suggested that taurolactone’s regulatory impact on AGGF1 could potentially restrict the proliferation and dissemination of HCC. The concurrent reduction in the expression levels of PDGF, Angiopoietin, and AGGF1 among patients in the observation group indicated that taurolactone could potentially disrupt HCC angiogenesis through multifaceted mechanisms.

The assessment conducted through MRI imaging techniques unveiled changes within the observation group. This included a decrease in both the mean and maximum blood flow in tumor blood vessels, along with a noteworthy prolongation of the peak time and arrival time of these vessels. These findings offered valuable insights into the impact of taurolactone on angiogenesis in HCC. Average perfusion, which is a measure of blood flow per unit volume, is often applied as an indicator of tumor blood supply [21]. The reduction in average perfusion among patients in the observation group indicated taurolactone’s effectiveness in diminishing blood flow to HCC tumors. This reduction in blood supply carries immense significance, as it limits the availability of nutrients and oxygen, thereby substantially impeding tumor growth. Maximum perfusion signifies the highest capacity of tumor blood vessels, typically associated with the hemodynamic properties of tumors [22, 23]. The observed reduction in maximum perfusion volume within the observation group indicated that taurolactone had a specific impact on suppressing vasodilation and enhancing blood flow in HCC tumors, thereby restricting their blood supply. The peak time is the moment when the contrast agent concentration in blood vessels reaches its maximum value, while the time of arrival signifies when the contrast agent first enters the tumor’s blood vessels. The prolongation of both peak time and arrival time in the observation group could potentially indicate alterations in angiogenesis mimicry within HCC. These changes may lead to an extended retention time of the contrast agent in blood vessels, thus reducing the blood flow velocity and blood supply to tumors. The alterations in tumor vascular perfusion parameters in the observation group indicated that taurolactone may exert its influence on the angiogenesis of HCC through multiple mechanisms.

A noteworthy discovery in this study pertains to tumor vascular density, a critical factor that deserves close attention. The findings indicated that taurolactone could potentially impact vascular density within HCC through its potent antiangiogenic properties. By directly targeting the growth of tumor vascular endothelial cells and the formation of new blood vessels, taurolactone reduced vascular density. The inhibition of signal pathways or growth factors of tumor vascular endothelial cells could potentially hinder their proliferation and the formation of new blood vessels in tumors. Tumor deterioration and growth restriction are often associated with this decline in tumor vascular density [24]. Reduced vascular density results in decreased oxygen and nutrient delivery, thereby restricting the survival and dissemination of cancerous cells [25]. This could trigger apoptosis (cell death) and metabolic dysfunction in cancer cells, ultimately decreasing their invasiveness and rate of growth. Furthermore, the extension of PFS and OS in the observation group underscored the potential of taurolactone to effectively hinder the progression of HCC. This extension in PFS and OS might be attributed to the various impacts of taurolactone on the biological process within HCC, including the suppression of angiogenesis, modulation of growth factors, and alterations in vascular perfusion. The findings demonstrated the possible benefits of taurolactone in enhancing patients’ survival and overall well-being.

In conclusion, the findings derived from this research underscored the capacity of taurolactone to significantly enhance the prognosis of individuals diagnosed with HCC by suppressing factors related to the formation of new blood vessels and imitating angiogenesis. This highlighted the substantial value of taurolactone in treating HCC. The findings from this investigation offered robust support for additional exploration and advancement of anti-cancer medications, thereby offering enhanced therapeutic alternatives for individuals diagnosed with HCC.

### Electronic supplementary material

Below is the link to the electronic supplementary material.

Supplementary Material 1

Supplementary Material 2

Supplementary Material 3

Supplementary Material 4

Supplementary Material 5

Supplementary Material 6

Supplementary Material 7

Supplementary Material 8

## Data Availability

The data and materials used and/or analysed during the current study are available from the corresponding author on reasonable request.

## Abbreviations

ANOVA: analysis of variance
DCE-MRI: dynamic contrast-enhanced magnetic resonance imaging
ELISA: enzyme-linked immunosorbent assay
HCC: hepatocellular carcinoma
PDGF: platelet-derived growth factor
