# Supplementary figures and images for: Effect of novel anti-tumor and anti-angiogenesis drug taurolactone on angiogenic factor AGGF1 and angiogenesis mimicry in patients with hepatocellular carcinoma
Source: BMC Cancer. 2024 May 21;24:614. doi: 10.1186/s12885-024-12356-w (PMC11106933; doi:10.1186/s12885-024-12356-w)

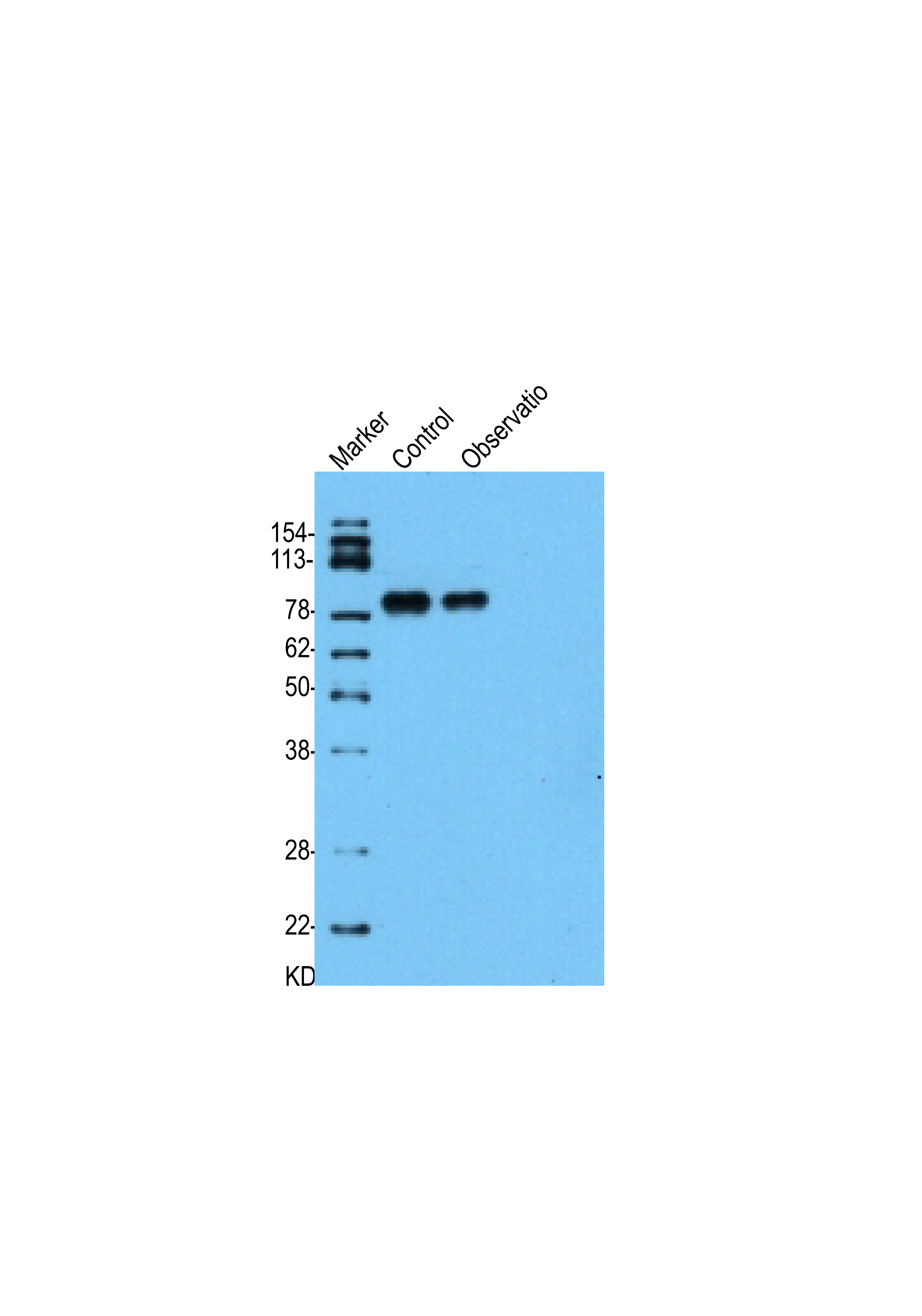

Supplement: Supplementary file 1 — Supplementary Material 1 [file 12885_2024_12356_MOESM1_ESM.tif]

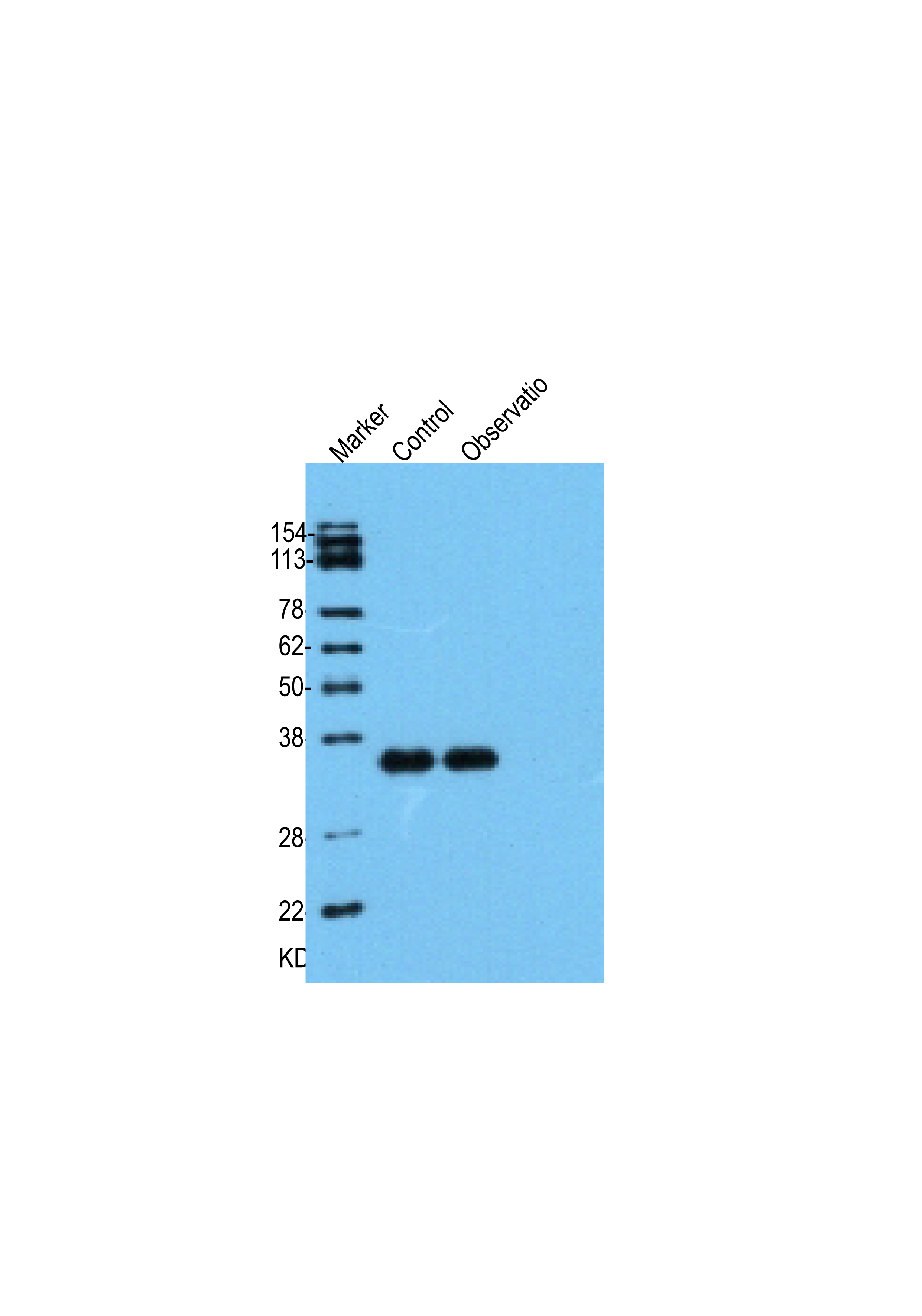

Supplement: Supplementary file 2 — Supplementary Material 2 [file 12885_2024_12356_MOESM2_ESM.tif]

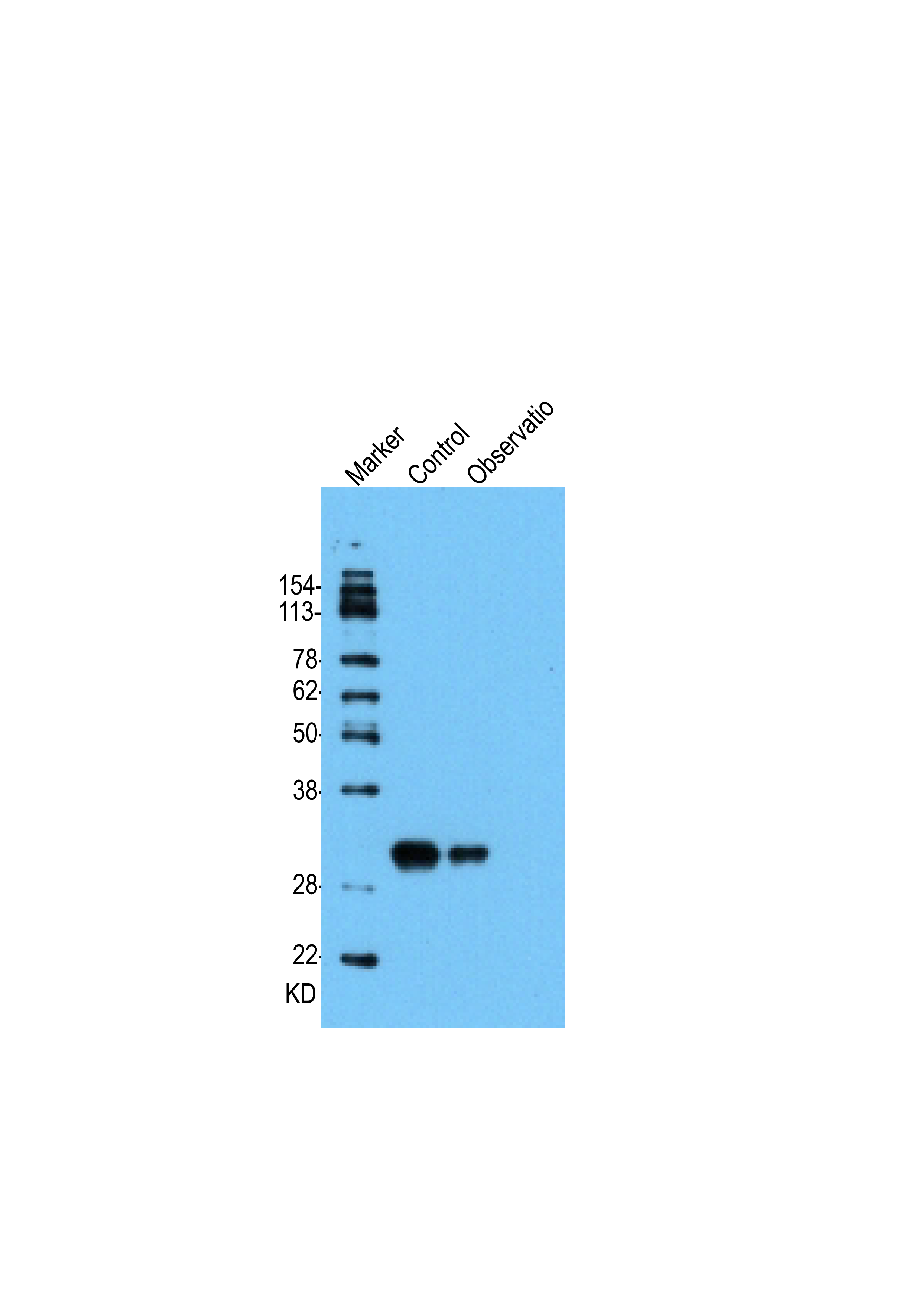

Supplement: Supplementary file 3 — Supplementary Material 3 [file 12885_2024_12356_MOESM3_ESM.tif]

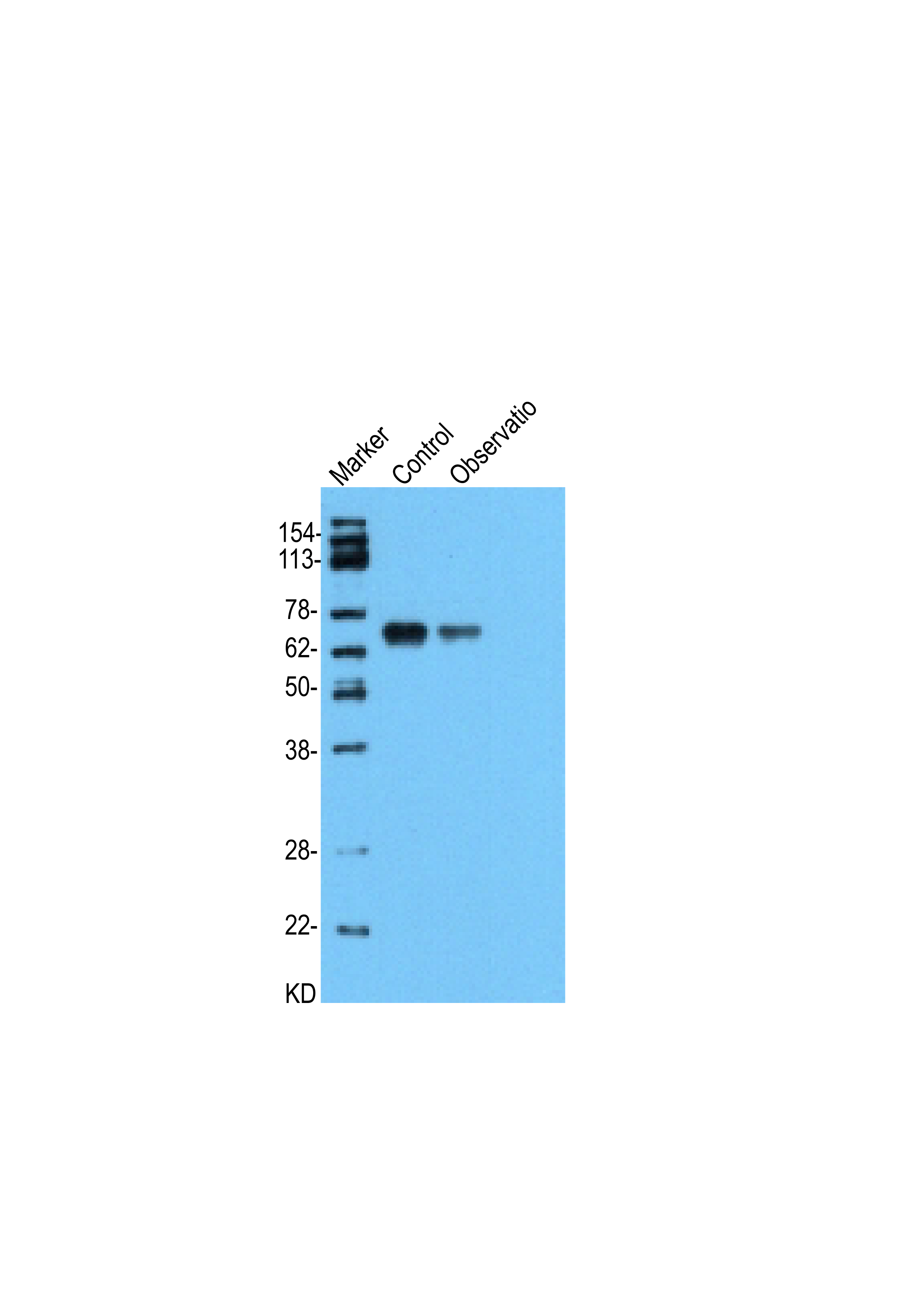

Supplement: Supplementary file 4 — Supplementary Material 4 [file 12885_2024_12356_MOESM4_ESM.tif]
